# Malignant pleural mesothelioma: Is reconstruction of the diaphragm necessary in left pleurectomy/decortication? A case report

**DOI:** 10.1002/ccr3.1973

**Published:** 2019-01-02

**Authors:** Alberto Testori, Matilde De Simone, Edoardo Bottoni, Marco Alloisio, Emanuele Voulaz, Ugo Cioffi

**Affiliations:** ^1^ Department of Thoracic Surgery Humanitas Research Hospital Milan Italy; ^2^ Department of Surgery University of Milan Milan Italy

**Keywords:** decortication, extrapleural pneumonectomy, malignant pleural mesothelioma, pleurectomy

## Abstract

We describe a case of complete intrathoracic stomach and intestinal herniation after pleurectomy/decortication due to diaphragm reconstruction without mesh. Is reconstruction with mesh always necessary? Can lung sparing obviate the tension on residual diaphragm? These are the questions not well described to which we try to give an answer.

## 1 INTRODUCTION

Over the last years, pleurectomy/decortication (P/D) has changed the surgical approach in the treatment of malignant pleural mesothelioma (MPM).^1^, ^2^

Extrapleural pneumonectomy (EPP) is a standardized procedure that needs three surgical times: exploration and removal of the pericardium and diaphragm, pneumonectomy, and reconstruction with meshes on diaphragm and pericardium.^3^

Pleurectomy/decortication is not well defined as EPP,^4^ first of all for the difference in performing the procedure between different surgeons; someone considering the procedure as a debulking procedure and other that try to use as radical surgery, then for the times of procedure itself especially regarding the removal and reconstruction of pericardium and diaphragm.^5^ Particularly, is the reconstruction necessary if you have got the preserved lung inside the chest?^6^

Here, we report a clinical case of diaphragm collapse with intrathoracic stomach and colon herniation after P/D.

## 2 CASE REPORT

A 50‐year‐old female, without comorbidity and with a previous histopathological confirmation of MPM obtained with pleural biopsy in video‐assisted thoracic surgery, received neoadjuvant chemotherapy (three cycles of carboplatinum and pemetrexed)^7^ and subsequently left P/D with complete removal of the tendineous part of the diaphragm with partial removal of muscle itself due to the direct extension of the illness. The residual diaphragm muscle was reconstructed with a synthetic monofilament continuous absorbable suture (Maxon™).^7^ During the first three postoperative days, the patient was normal, no bleeding, with normal chest X‐ray, and normal air leaking as common in P/D procedures in our experience. In the IV postoperative day, she stopped air leaking and referred chest pain and dyspnea.

**Figure 1:**
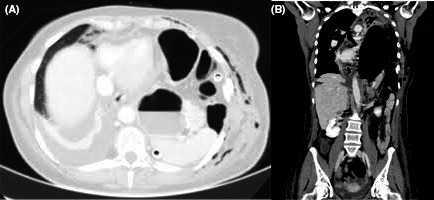
A,B, CT view of intrathoracic intestinal herniation

**Figure 2:**
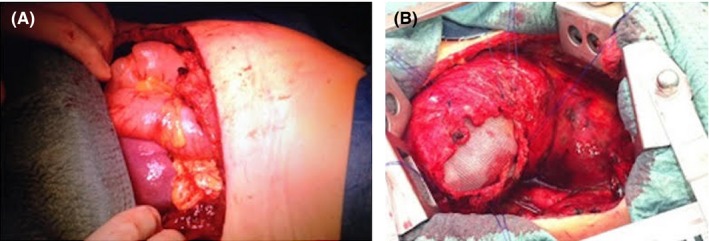
A,B, View of intrathoracic intestinal herniation and diaphragm reconstruction with the polypropylene mesh positioned under the diaphragm

At the chest X‐ray, there was evidence of complete collapse of the lung and air image resembling the stomach. A chest CT scan showed complete intrathoracic stomach and intestinal herniation (Figure 1A,B). After rethoracotomy, the complete herniation of the stomach and bowel was observed, with the spleen near the heart. To avoid other complication, as infection, we used a polypropylene mesh under the diaphragm and fixed it to the abdominal side of the diaphragm. Then, we made the reconstruction of the residual diaphragm muscle over the mesh. At the end of the reconstruction, we had the mesh not in contact with the lung and with the residual air leaks (Figure 2A,B) but on the abdominal side of the muscle. A nasogastric tube was positioned to maintain clean and decompressed the stomach.

The patient was discharged after ten postoperative days, without problems and with complete resolution of the herniation.

## 3 DISCUSSION

Malignant pleural mesothelioma is an aggressive neoplasm with no efficacy treatment until today. The best option is represented by trimodal approach: chemotherapy, surgical procedure (extrapleural pneumonectomy or pleurectomy/decortications), and radiation therapy; but also in these cases the life expectation is extremely poor, with median survivor of 24‐months in large series. After this surgical complication, the question if doing diaphragm reconstruction after left P/D remains controversial. During the last 8 years, we have performed about 70 cases of P/D and we have never recorded a diaphragmatic damage that required mesh positioning; this case report is the first in which the diaphragm breach was observed with bowel herniation. In literature, there is no clear evidence about diaphragm reconstruction during P/D and it depends by the surgeons and obviously on the volume of demolition of the muscle. After this case, we are not sure if P/D is a correct surgical technique without mesh positioning on the diaphragm, and if our complication depends only from the extension of muscle demolition. So the question arises spontaneously; is reconstruction of the diaphragm with meshes always necessary?^8^ In our series, the diaphragm reconstruction with mesh was done only when the muscle was completely removed. We do this to avoid infection due to synthetic materials in surgical operation that ever have prolonged air leaks.

## 4 CONCLUSION

Then, is reconstruction really necessary?^8^ After this complication, we believe that the mesh reconstruction is really necessary for the left P/D. However, one thing is certain that chest x‐rays should be performed every postoperative days until the patient is discharged because normal blood tests do not allow to see early variation in the chest.

## CONFLICT OF INTEREST

None declared.

## AUTHORS’ CONTRIBUTIONS

AT and EV: performed the operation, carried out the study, revised the manuscript. EB and MDS: helped in drafting the manuscript and revised the contents of the discussion of the manuscript. UC: carried out the concept and the design of the study and revised the manuscript.

## ETHICS APPROVAL AND CONSENT TO PARTICIPATE

Not applicable.

## CONSENT FOR PUBLICATION

Written informed consent was obtained from the patient for publication of this Case report and any accompanying images. A copy of the written consent is available on request.

## References

[1] Cao C , Tian D , Park J , Allan J , Pataky KA , Yan TD . A systematic review and meta‐analysis of surgical treatments for malignant pleural mesothelioma. Lung Cancer. 2014;83:240–245. 24360321 10.1016/j.lungcan.2013.11.026

[2] Flores RM , Pass HI , Seshan VE , et al. Extrapleural pneumonectomy versus pleurectomy/decortication in the surgical management of malignant pleural mesothelioma: results in 663 patients. J Thorac Cardiovasc Surg. 2008;135; 620–626. 626.e1–3. 18329481 10.1016/j.jtcvs.2007.10.054

[3] Sugarbaker DJ , Mentzer SJ , Strauss G . Extrapleural pneumonectomy in the treatment of malignant pleural mesothelioma. Ann Thoracic Surg. 1992;54:941–946. 10.1016/0003-4975(92)90654-m 1417290

[4] Lang‐Lazdunski L , Bille A , Lai R , et al. Pleurectomy/decortication is superior to extrapleural pneumonectomy in the multimodality management of patients with malignant pleural mesothelioma. J Thorac Oncol. 2012;7:737–743. 22425923 10.1097/JTO.0b013e31824ab6c5

[5] Bassuner JK , Rice DC , Antonoff MB , et al. Polytetrafluoroethylene or acellular dermal matrix for diaphragmatic reconstruction? Ann Thorac Surg. 2017;103:1710–1714. 28366460 10.1016/j.athoracsur.2017.01.006

[6] Flores RM . Induction chemotherapy, extrapleural pneumonectomy, and radiotherapy in the treatment of malignant pleural mesothelioma: the Memorial Sloan‐Kettering experience. Lung Cancer. 2005;49(Suppl 1):S71–S74. 15950805 10.1016/j.lungcan.2005.03.015

[7] Kobayashi H , Nomori H , Mori T , Shibata H , Yoshimoto K , Ohba Y . Extrapleural pneumonectomy with reconstruction of diaphragm and pericardium using autologous materials. Ann Thorac Surg. 2009;87:1630–1632. 19379936 10.1016/j.athoracsur.2008.09.068

[8] De Perrot M , McRae K , Anraku M , et al. Risk factors for major complications after extrapleural pneumonectomy for malignant pleural mesothelioma. Ann Thorac Surg. 2008;85:1206–1210. 18355497 10.1016/j.athoracsur.2007.11.065

